# Coronary Flow Reserve of the Non-Ischemia Related Coronary Artery During Dobutamine Stress Echocardiography

**DOI:** 10.4021/cr57w

**Published:** 2011-07-25

**Authors:** Dawod Sharif, Amal Sharif-Rasslan, Camilia Shahla, Amin Khalil, Uri Rosenschein

**Affiliations:** a Department of Cardiology, Bnai Zion Medical Center, Haifa, Israel; b Technion – Israel Institute of Technology, Haifa, Israel

**Keywords:** Dobutamine stress echocardiography, Coronary flow reserve, Myocardial ischemia

## Abstract

**Background:**

Incorporation of analysis of coronary velocities in stress studies adds diagnostic value to both clinical variables and dobutamine echocardiography. Micorcirculatory abnormalities may precede obstructive corornary disease. Therefore the aim of this study was to assess Doppler derived coronary velocity and flow of the left anterior descending coronary artery (LAD) during dobutamine stress echocardiography (DSE) in patients without LAD-related ischemia.

**Methods:**

Sixty nine patients with chest pain underwent DSE studies to evaluate myocardial ischemia. All had trans-thoracic Doppler interrogation of the distal LAD before and just after termination of the DSE. Coronary velocity reserves (CFR) were calculated as the ratios of post-DSE/baseline diastolic velocities. Volumetric flow in the distal LAD was calculated from the diameter of LAD color jet and velocity integral. Volumetric flow reserve was calculated as the ratio of post-DSE baseline LAD flows.

**Results:**

At rest all subjects had left ventricular wall motion score index (WMSI) = 1, while in 28, wall motion abnormality appeared in non-LAD territory with WMSI = 1.17 ± 0.08. Peak diastolic velocity after DSE increased form 28.5 ± 13.6 to 52.4 ± 23.7 cm/sec, P = 9.5 × 10^-11^, and velocity-CFR was 2.08 ± 0.7. Diastolic LAD flow increased from 36.5 ± 23.8 to 75.75 ± 48.7 mL/min, P = 1.21 × 10^-7^ and volumetric-CFR was 2.6 ± 2.8. Peak diastolic velocity-CFR in patients without LV wall motion abnormality was 2.4 ± 0.7 while in those with motion abnormality 1.77 ± 0.56, P = 0.00008. Flow-derived LAD-CFR was 3.3 ± 3.7 in those without compared to 1.88 ± 0.57 in patients with wall motion abnormality, P < 0.05.

**Conclusion:**

LAD velocity and flow reserves are reduced in patients with remote myocardial ischemia, which may indicate early atherosclerotic involvement.

## Introduction

Dobutamine stress echocardiography (DSE) is well established in the assessment of coronary artery disease (CAD) and relies mainly on changes in radial motion and thickening of left ventricular myocardial segments [1, 2]. Recently, sampling of coronary artery velocities especially of the left anterior descending coronary artery by transthoracic Doppler became feasible [3-7]. Incorporation of analysis of coronary velocities in stress studies adds diagnostic value to both clinical variables and dobutamine echocardiography [8]. Thus, coronary flow reserve of the left anterior descending coronary artery is more sensitive and less specific than analysis of wall motion abnormalities [9], and the diagnostic accuracy of both methods are similar [10]. Evaluation of coronary flow reserve depends on the baseline velocities and hyperemic velocities after stress. Coronary velocities may be affected by other variables in addition to stress. The aim of the present study was to evaluate whether presence of remote myocardial ischemia affects coronary flow reserve in the non-ischemic left anterior descending coronary artery.

## Methods

### Population

Sixty nine patients, age 66 ± 8 years, underwent DSE studies to evaluate CAD, all had no left ventricular wall motion abnormality at rest and without history of myocardial infarction.

### Dobutamine stress echocardiography

The protocol of dobutamine infusion consisted of 3 minute stages for each dose, starting with 5 µg/kg/min and increasing to 10, 20, 30 and 40 µg/kg/min. If end-points did not occur or 85% of the age adjusted heart rate was not achieved, 0.25 mg atropine was injected every 2 minutes up to 1 mg or until the target heart rate was achieved. Blood pressure and 12 lead electrocardiograms were recorded at rest and throughout the DSE study. Horizontal or down-sloping > 1 mm ST-segment depression at 0.06 sec after the J point were considered as evidence for myocardial ischemia.

### Image acquisition

Images were obtained while the patients in the left lateral decubitus position. A standard commercial Acuson Sequoia echocardiographic system equipped with 3.5-7 MHZ transducer was used. Parasternal long axis and short axis as well as apical 4-chamber and 2-chamber views were recorded at rest, low dose dobutamine infusion, peak exercise and in the recovery period. Digital images were stored on magneto-optic discs for later off-line analysis. In addition super VHS videotape recordings were performed throughout the studies.

### Dobutamine stress echocardiographic analysis

Segmental left ventricular wall motion analysis was performed using 16-segment model [11]. Regional wall motion was estimated and scored as normal = 1, hypokinetic = 2, akinetic = 3 and dyskinetic = 4. Analysis of the gray-scale 2-dimensional images were used for the assessment of regional left ventricular wall motion. New or worsening segmental wall motion was considered as ischemic response. Ischemic response (I) was identified when wall motion decreased by at least 1 grade in 2 adjacent segments or wall motion decreased by at least 2 grades in 1 segment, otherwise no ischemia, or normal response (N) was diagnosed. Left ventricular wall motion score index (WMSI) was calculated as: LV-WMSI = ∑ (score of 16 segments) / 16.

### Velocities of the left anterior descending coronary artery

In order to obtain LAD flows the color Doppler Nyquist limit was set at 17 cm/sec. Systematic attempt to get LAD-color flow were performed. From low parasternal short axis view, search for diastolic color flow in the anterior interventricular groove followed by clockwise rotation performed, while form apical foreshortened two chamber views LAD diastolic flow was located in the interventricular groove and the counterclockwise rotation of the transducer was performed.

### Measurements

**Figure 1:**
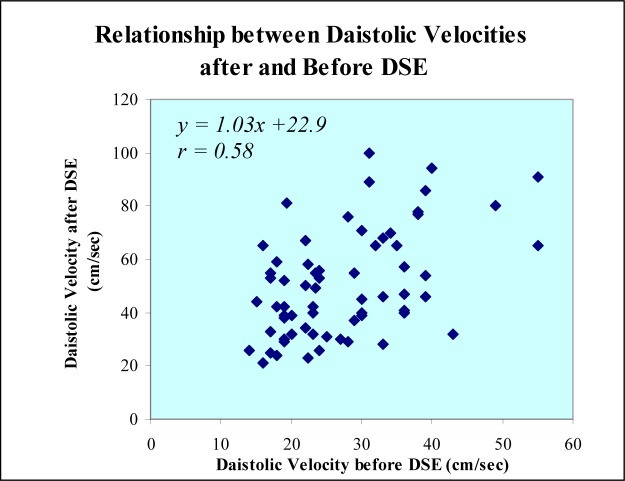
Scattergram of diastolic left anterior descending coronary artery (LAD) velocity before and after dobutamine stress echocardiography (DSE).

Peak diastolic (VD) and systolic velocities (VS) and their ratios VD/VS were performed. In addition time velocity integrals (TVI) in diastole (TVID) and systole (TVIS) and their ratios (TVID/TVIS) were measured. Diameter of LAD was measured form color Doppler velocity profile (Fig. 1) and LAD cross sectional area was calculated as: Area = π (diameter (cm))^ 2^ / 4. Flow in the LAD was calculated as: Flow = (Heart Rate) × (LAD - Area) × TVI. For diastolic flow TVID and for systolic flow TVIS were used, their sums and ratios were also calculated. All had transthoracic Doppler interrogation of the distal LAD velocities as described previously. Sampling of LAD velocities were performed at baseline, and just after termination of the DSE. Coronary flow reserves (CFR) were calculated as the ratios of post-DSE/baseline diastolic velocities, as well as post-DSE/baseline LAD flows.

### Statistical analysis

Mean values and standard deviations of measurements were calculated. Two-tailed student t-test was used and P < 0.05 was considered significant. Measurements were compared before and after DSE. A comparison between normal DSE-group and the group with ischemia not in the LAD territory was performed.

## Results

All subjects underwent dobutamine stress echocardiography safely without events. Heart rate increased from 68.12 ± 17.3 at rest to 131.8 ± 16.4 bpm after stress echocardiography. Systolic blood pressure increased from 134 ± 11 mmHg to 164 ± 14 mmHg. Sampling of left anterior descending coronary artery velocities was possible in all.

### Left ventricular wall motion

All 69 subjects had no left ventricular wall motion abnormality at rest. After dobutamine, left ventricular wall motion abnormality (hypokinesis and akinesis), appeared in 28 subjects, WMSI = 1.17 ± 0.08; none in left anterior descending coronary artery territory. Coronary angiography revealed coronary steonsis in non-LAD arteries. In the remaining subjects, WMSI remained 1 after DSE.

### Left ventricular wall motion and patient characteristics

In subjects with left ventricular wall motion abnormality at rest or during stress compared to those without, coronary artery disease risk factors included arterial hypertension in 30.4% and 26.1%, hyperlipidemia 27.5% and 23.2%, cigarette smoking 24.6% and 23.2%, obesity 14.5% and 15.9%, family history 11.6% and 10.2%, diabetes mellitus in 8.7% and 8.7% respectively, without significant differences between the groups.

### Doppler velocities through the left anterior descending coronary artery

Diastolic velocities, time velocity integrals and diastolic flows through the LAD increased significantly after DSE compared to values at rest, table 1. In addition, calculated LAD systolic flows and total flows (sum of systolic and diastolic flows) increased significantly after DSE (Table 1). The increase in velocities was present in the whole study population, in those with and those without wall motion abnormality.

**Table 1:** LAD Parameters Before and After DSE

		VD (cm/sec)	TVID (cm)	Diastolic Flow (mL/min)	Systolic Flow (mL/min)	Total Flow (mL/min)
All subjects (n = 69)	Rest	28.5 ± 13.6	9 ± 3.8	36.5 ± 23.8	11.6 ± 12.2	47.6 ± 34.8
	DSE	52.4 ± 23.7	10.5 ± 4.7	75.75 ± 48.7	23.8 ± 22.7	99.8 ± 68.2
	P-value	9.5 × 10^-11^	0.04	1.21×10^-7^	0.000242	4.58 ´ 10^-7^
With WMA (n = 41)	Rest	33 ± 18	10 ±4.8	41 ± 29.7	12.6 ± 15	53.6 ± 43
	DSE	56.7 ± 29	11 ±5.8	75.1 ± 55	24.4 ± 22.7	99.5 ± 74
	P-value	0.0008	0.47	0.009	0.035	0.01
Without WMA (n = 28)	Rest	25.5 ± 8.6	8.23 ±2.9	33.5 ± 18.7	11 ± 10	43.6 ± 28.4
	DSE	49.5 ± 19.2	10.1 ±3.9	76 ± 44.7	23.88 ± 23.3	100 ± 65.3
	P-value	1.17 × 10^-9^	0.015	1.72 × 10^-6^	0.00289	1.05 × 10^-5^

Value: mean ± SD. SD: standard deviation.

Diastolic left anterior descending coronary artery blood velocities after stress echocardiography correlated positively with baseline velocities (Fig. 1).

### Coronary flow reserve in the left anterior descending coronary artery

Coronary flow reserve in subjects with left ventricular wall motion abnormality was lower than in those without (Table 2).

**Table 2:** LAD-CFR Parameters in All Subjects

	CFR VD	CFR VS	CFR TVID	CFR TVIS	CFR Diastolic Flow	CFR Systolic Flow	CFR Total Flow
All subjects	2.08 ± 0.7	2.04 ± 1.5	1.23 ± 0.4	1.45 ± 1.09	2.6 ± 2.8	2.9 ± 3.3	2.57 ± 2.79
With Ischemia	1.77 ± 0.56	1.4 ± 0.56	1.13 ± 0.3	1.48 ± 1.35	1.88 ± 0.57	2.6 ± 2.4	1.97 ± 0.66
Without Ischemia	2.4 ± 0.7	2.45 ± 1.78	1.3 ± 0.55	1.4 ± 0.89	3.3 ± 3.7	3.13 ± 3.7	2.98 ± 3.5
P- value	0.0008	0.001	0.12	0.84	0.045	0.5	0.09

Value: mean ± SD. SD: standard deviation.

**Figure 2:**
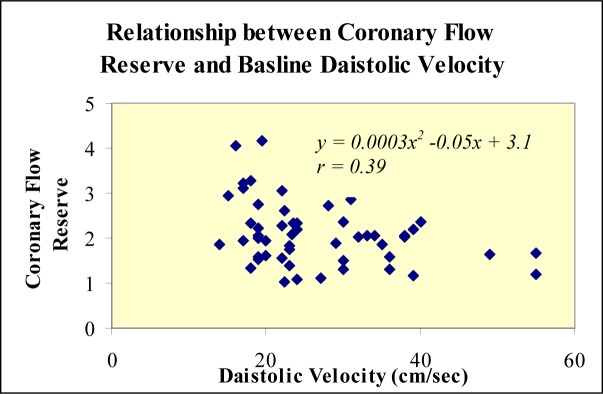
Scattergram of coronary flow reserve of the left anterior descending coronary artery as a function of diastolic velocity at rest.

**Figure 3:**
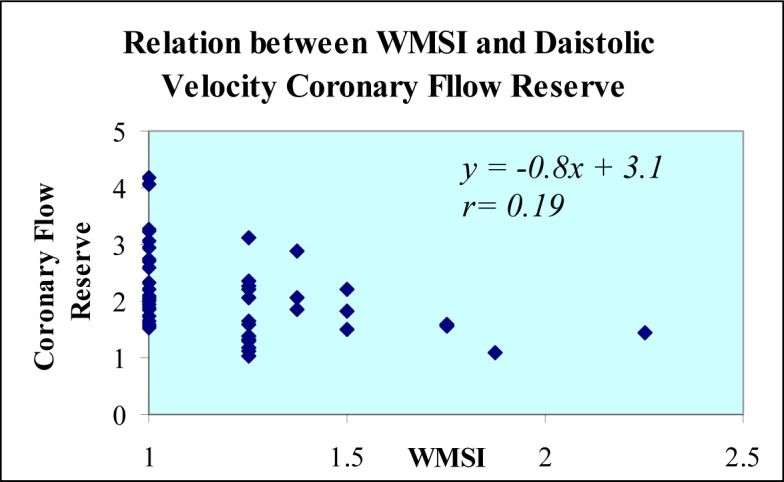
Scattregram of coronary flow reserve of the left anterior descending coronary artery as a function of wall motion score index (WMSI) after dobutamine stress echocardiography.

Coronary flow reserve of left anterior descending coronary artery tended to be lower in those with higher baseline diastolic velocities, (Fig. 2). In addition, the larger the left ventricular wall motion score index, the lower the coronary flow reserve (Fig. 3).

## Discussion

In the present study, administration of dobutamine induced an increase in velocities and flows in the non-ischemia related LAD with or without remote regions of wall motion abnormalities. In subjects with left ventricular wall motion abnormalities in non-LAD territories, LAD velocities and flows increased less than in the other group, and thus had significantly lower coronary flow reserves.

Coronary artery stenosis decreases microvascular flow reserve in the stenotic bed [12]. Evaluation of coronary flow reserve was applied during stress echocardiography with dobutamine solely or with the addition of adenosine before the stress for the assessment of coronary artery disease [13, 14]. Results of the present study imply that coronary stenosis is associated with reduction of coronary flow reserve in remote non-stenontic beds, as was shown previously in both experimental animal models and in humans [15-20]. Our findings are also in accord with previous studies showing that in patients with stable angina pectoris, coronary flow reserve is abnormal in angiographically normal coronary arteries [21, 22]. Moreover, patients with acute myocardial infarction may have reduced flow in the non-infarct related artery, severe enough to be associated with regional wall motion abnormality remote from the infracted area [23].

Coronary endothelial dysfunction involving the non-ischemia related LAD may contribute to the findings of the present study. Endothelial dysfunction precedes other manifestations of atherosclerosis, e.g. cigarette smokers have brachial [24] and coronary flow [25] abnormalities which may normalize after the administration of vitamin C. Asymptomatic subjects with hyperlipidemia have potentially reversible coronary flow abnormalities [26-29]. Patients with diabetes mellitus have abnormal coronary flow and flow reserve [30-32].

Effects of dobutamine on sympathetic receptors of the LAD may contribute to the results found in this study. Clinically available dobutamine is a racemic mixture that stimulates both beta-1 and beta-2 adrenergic receptors [33]. Dobutamine is nonselective in binding to beta-1 and beta-2 receptors, but binding of each isomer to alpha receptors may result in both agonist and antagonist activity [34] and at higher doses exerts alpha1-adrenergic agonist action. Thus, in patients with coronary artery disease, coronary flow in angiographically normal coronary artery may be affected by endothelial function and by the effects of dobutamine on the coronary arteries and the myocardium which may be reversed by alpha-blocking agents [34].

Evaluation of coronary flow reserve may be applied in coronary arteries with intermediate lesions to decide on the need for intervention. Moreover, now that transthoracic sampling of coronary velocities and especially of the LAD became possible, evaluation of coronary flow reserve may be incorporated in the evaluation for coronary artery disease. The results of the present study suggest that different criteria may be needed in the presence of wall motion abnormality in the area without apparent ischemia.

### Study limitations

Coronary atherosclerosis may be detected by intracoronary ultrasound and recently by noninvasive multi-detector computed coronary artery angiography, which were not performed in this study; thus coronary flow reserve of the left anterior descending coronary artery could not be related to the presence of atherosclerosis in this vessel.

### Summary and clinical implications

Sampling of LAD velocities and calculations of flow and CFR are feasible before and just after DSE. LAD velocities and flows increase during DSE. Coronary flow reserve of the LAD is reduced in subjects with remote, non-LAD related ischemia compared to normal subjects. Incorporation of evaluation of CFR of the LAD during DSE is feasible, but presence of reduced CFR may indicate functional abnormality without significant anatomic stenosis.
